# Post-translational regulation enables robust p53 regulation

**DOI:** 10.1186/1752-0509-7-83

**Published:** 2013-08-30

**Authors:** Yong-Jun Shin, Kai-Yuan Chen, Ali H Sayed, Brandon Hencey, Xiling Shen

**Affiliations:** 1 School of Electrical and Computer Engineering, 402 Phillips Hall, Cornell University, Ithaca, NY 14853, USA; 2 Department of Electrical Engineering, University of California, Los Angeles, CA 90095-1594, USA; 3 School of Mechanical and Aerospace Engineering, 214 Upson Hall, Cornell University, Ithaca, NY 14853, USA

**Keywords:** Feedback control theory, p53-Mdm2 feedback loop, Robustness, Disturbance rejection

## Abstract

**Background:**

The tumor suppressor protein p53 plays important roles in DNA damage repair, cell cycle arrest and apoptosis. Due to its critical functions, the level of p53 is tightly regulated by a negative feedback mechanism to increase its tolerance towards fluctuations and disturbances. Interestingly, the p53 level is controlled by post-translational regulation rather than transcriptional regulation in this feedback mechanism.

**Results:**

We analyzed the dynamics of this feedback to understand whether post-translational regulation provides any advantages over transcriptional regulation in regard to disturbance rejection. When a disturbance happens, even though negative feedback reduces the steady-state error, it can cause a system to become less stable and transiently overshoots, which may erroneously trigger downstream reactions. Therefore, the system needs to balance the trade-off between steady-state and transient errors. Feedback control and adaptive estimation theories revealed that post-translational regulation achieves a better trade-off than transcriptional regulation, contributing to a more steady level of p53 under the influence of noise and disturbances. Furthermore, post-translational regulation enables cells to respond more promptly to stress conditions with consistent amplitude. However, for better disturbance rejection, the p53- Mdm2 negative feedback has to pay a price of higher stochastic noise.

**Conclusions:**

Our analyses suggest that the p53-Mdm2 feedback favors regulatory mechanisms that provide the optimal trade-offs for dynamic control.

## Background

Gene networks are constantly subject to noise or fluctuations, which originate from variations in transcription, translation, and environmental conditions. The stochastic nature of gene networks has been the focus of many studies (reviewed in [1,2]). There are at least three types of fluctuations that affect gene network dynamics: 1) intrinsic noise or fast fluctuations, 2) extrinsic noise or slow fluctuations, and 3) periodic DNA replication-dependent oscillations [3]. Intrinsic noise arises from the inherent randomness during transcription and translation, key processes for gene expression [4]. Extrinsic noise arises from the factors that universally affect the expression of all genes in a given cell, such as variations in the number of RNA polymerase, ribosome, etc. [1,2]. The third type of fluctuation is due to periodic DNA replication in growing and dividing cells [3].

Nevertheless, gene networks are usually able to perform their regulatory functions under the influence of such disturbances [5], which provokes the question: How do they manage to achieve this remarkable robustness? In control theory, it is known that feedback, a situation in which two (or more) dynamical sub-systems are connected in a way that their dynamics are coupled, can make a system resilient towards disturbances [6,7]. A well-known example of feedback in the context of gene networks is negative auto-regulation, in which a transcription factor represses the transcription of its own gene and reduces the effects of noise exerted on the transcription process [8-10].

**Figure 1:**
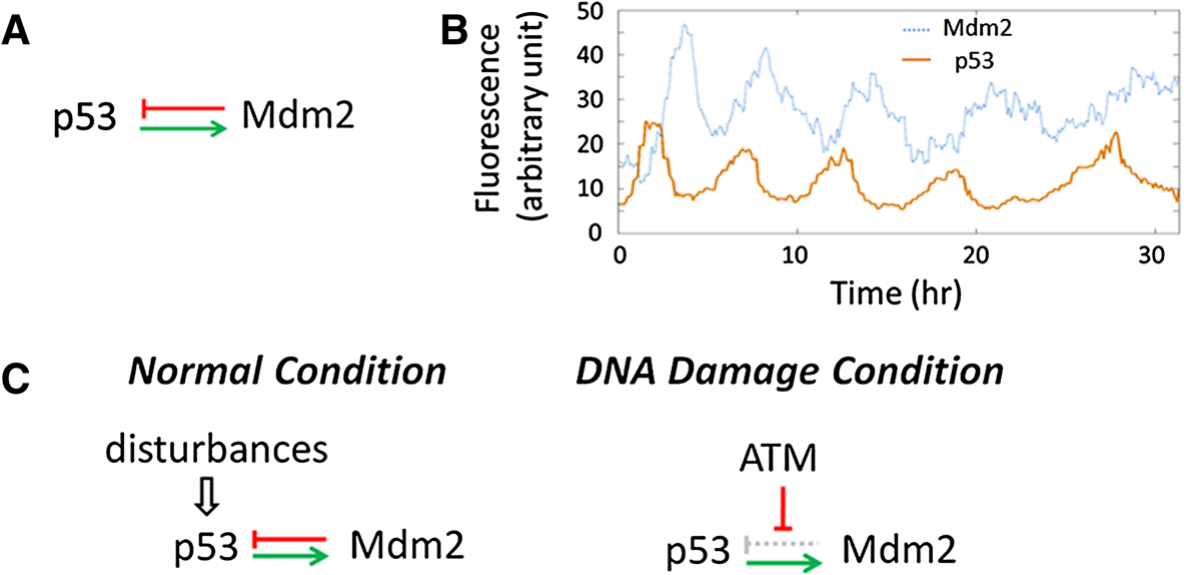
**The p53-Mdm2 Feedback Loop. (A)** p53 transcriptionally activates Mdm2, while Mdm2 degrades p53. **(B)** The p53 and Mdm2 levels oscillate upon DNA damage [21]. **(C)** Under normal conditions, Mdm2 is not suppressed by ATM and the intact feedback rejects disturbances. Upon DNA damage when p53 oscillates, the feedback is suppressed to allow p53 oscillation.

Another example is the p53-Mdm2 negative feedback, in which p53 transcriptionally activates Mdm2, an E3 ubiquitin ligase, while Mdm2 targets p53 for degradation (Figure 1A) [11]. As one of the most studied tumor suppressor proteins [12,13], p53 plays a key role in repairing DNA damage, arresting cell cycle and, when damage is beyond repair, activating apoptosis (programmed cell death) [14,15]. Therefore, it is important for the cell to regulate p53 robustly, because disturbances may trigger unwanted cell cycle arrest or apoptosis. We have previously shown that the p53-Mdm2 negative feedback can reject disturbances and improve robustness under normal (non-stressed) conditions [16]. Intuitively, the negative feedback tries to compensate for changes in the p53 level, so that the impact of any disturbances is offset or at least attenuated.

The feedback is suppressed to stop disturbance rejection during DNA-damage. Within minutes of exposure to DNA-damaging agents (UV, X-rays, etc.), the p53 protein level increases rapidly without any significant change in the p53 mRNA level, for p53 is stabilized and no longer degraded by Mdm2 at the normal rate [17,18]. The suppression of the feedback and the stoppage of disturbance rejection allow external factors such as ATM to modulate p53 in response to stress [19,20], which can result in pulses or sustained oscillation of p53 [21] (Figure 1B). Therefore, the feedback mechanism is adaptive – under normal conditions the feedback rejects disturbance to maintain a low steady level of p53 whereas upon DNA damage, the feedback is inactivated to allow pulses or oscillation [16] (Figure 1C).

Interestingly, even though the negative feedback is stronger in normal conditions to reject disturbances, it is well known that feedback can also cause instability, which leads to transient fluctuation (overshoot) and oscillation [7]. Both the strength and the delay of a negative feedback can contribute to instability. As previous measurements showed, the transcriptional regulation of Mdm2 by p53 has a noticeable delay [21] (Figure 1B). Therefore, to maintain a steady p53 level in normal conditions, the p53-Mdm2 feedback faces a dilemma: the stronger the feedback is to reject disturbances at the steady state, the more likely the feedback will become unstable and cause transient or sustained fluctuations. The feedback has to carefully balance the trade-off between steady-state and transient errors for disturbance rejection [22].

Does the p53-Mdm2 feedback adopt any strategy to optimize this trade-off? It is intriguing that in this negative feedback loop Mdm2 downregulates p53 through a post-translational mechanism (protein degradation), which is not as energy efficient as transcriptional repression because p53 is being produced and actively degraded simultaneously. However, post-translational regulation has distinct dynamic properties and is relatively faster than transcriptional regulation, so it is conceivable that post-translational regulations provides an advantage over transcriptional regulation in terms of the robustness-stability trade-off despite its less energy efficiency. To test this hypothesis, we used techniques from feedback control and adaptive estimation theories to analyze the p53-Mdm2 feedback loop.

## Results and Discussion

### p53-Mdm2 feedback model

To study the p53-Mdm2 feedback, we started with a previously published p53-Mdm2 feedback model that matches experimental measurements [23] and added a term representing ATM (Eqs. 1–3):

(1) $$\frac{\mathrm{dx}\left(t\right)}{\mathrm{dt}}=0$$

(2) $$\frac{\mathrm{dy}\left(t\right)}{\mathrm{dt}}=p_{\mathrm{zy}}z\left(t\right)-p_{\mathrm{xy}}x\left(t\right)-p_{y}y\left(t\right)$$

(3) $$\frac{\mathrm{dz}\left(t\right)}{\mathrm{dt}}=-p_{\mathrm{yz}}y\left(t\right)-p_{z}z\left(t\right)\;$$

where *x(t)*, *y(t)*, and *z(t)* represent ATM, Mdm2, and p53 levels, respectively. In Eq. 1, since ATM is inactive under normal conditions, the ATM levels are assumed to be low and constant and the rate of change is zero. Eq. 2 is composed of the p53-dependent production of Mdm2 (first term), ATM-dependent suppression of Mdm2 (second term), and Mdm2 degradation (third term). Eq. 3 is composed of the Mdm2-dependent suppression of p53 (first term) and p53 degradation (second term). Note that all the parameter values are supposed to be positive. Following the practice in the published model [23], Eq. 3 does not include the constant basal production rate because it has no effect on the frequency domain and disturbance rejection analysis we will perform next [23]. A discrete-time model for the p53-Mdm2 feedback system was also built for parameter estimation, which will be described in later sections of the paper.

### Feedback reduces steady-state error

Eq. 3 is rather generic and can represent alternative p53 suppression mechanisms by Mdm2. A transcriptional mechanism would only affect *p*_*yz*_ in the first term because the strength of transcriptional suppression only depends on the number of Mdm2 molecules (suppressor) but not on the number of p53 protein molecules. In contrast, a post-translational mechanism would affect both *p*_*yz*_ (first term) and *p*_*z*_ (second term), because the degradation rate of p53 depends on both the number of p53 molecules and the number of Mdm2 molecules. This generic equation enables us to derive a common set of equations to compare the loop dynamics between transcriptional and translational regulation.

**Figure 2:**
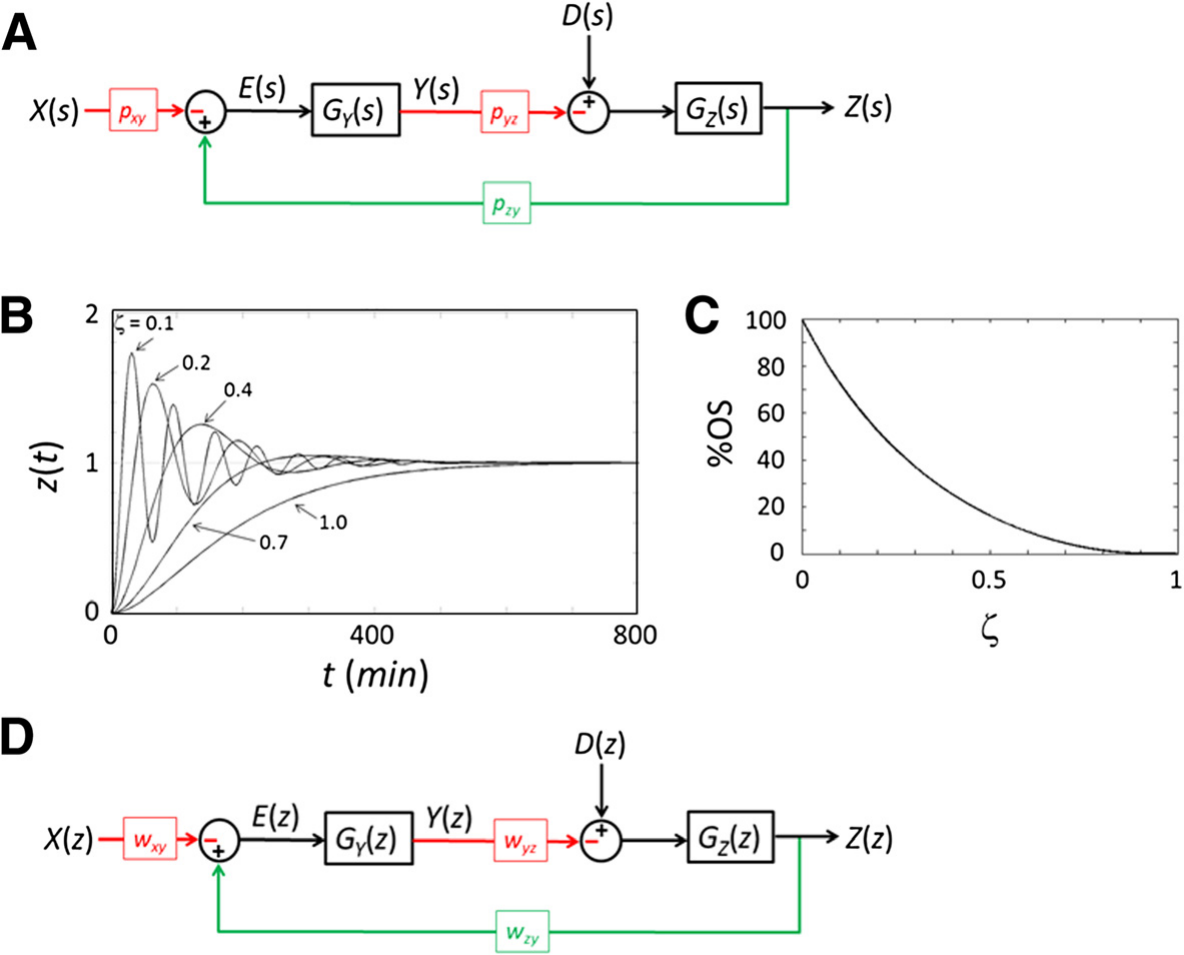
**Feedback analysis. (A)** Block diagram representation of the feedback in Eqs. 1–3. *X*(*s*), *Y*(*s*) and *Z*(*s*) are the Laplace transforms of *x*(*t*), *y*(*t*), and *z*(*t*). *G*_*Y*_(*s*) and *G*_*Z*_(*s*) are the transfer functions. *E*(*s*) is the Laplace transform of the error *e*(*t*) between the input and output. *D*(*s*) represents the disturbance. **(B)** Time responses (unit step response) for a second-order system with different damping ratios (*p*_*z*_ = *p*_*y*_ = 0.01 min^-1^). **(C)** As the damping ratio (ζ) increases from 0 to near 1 the %OS decreases from 100% to near 0%. **(D)** Block diagram representation of the discrete-time feedback model. *X*(*z*), *Y*(*z*), and *Z*(*z*) are the z-transforms of *x*(*i*), *y*(*i*), and *z*(*i*). *G*_*Y*_(*z*) and *G*_*Z*_(*z*) are the transfer functions. *E*(*z*) is the transform of the error *e*(*i*) between the input and output. *D*(*z*) represents the disturbance.

Eqs. 1–3 can be represented as a block diagram using the Laplace transform [24] (Figure 2A). *X*(*s*), *Y*(*s*), and *Z*(*s*) denote the Laplace transforms of *x*(*t*), *y*(*t*), and *z*(*t*) respectively. *E*(*s*) is the difference, or error, between the input, *p*_*xy*_*X*(*s*), and the output, *p*_*zy*_*Z(s*). *D*(*s*) represents any disturbance exerted on p53. *G*_*Y*_(*s*) and *G*_*Z*_(*s*) are the transfer functions that represent the Mdm2 and p53 systems as we showed previously [24]:

(4) $$G_{Y}\left(s\right)=\frac{1}{s+p_{y}},\;G_{Z}\left(s\right)=\frac{1}{s+p_{z}}$$

From Figure 2A, *E*(*s*) can be expressed as [16]:

(5) $$E\left(s\right)=Z\left(s\right)p_{\mathrm{zy}}-X\left(s\right)p_{\mathrm{xy}}\to \;Z\left(s\right)=\frac{E\left(s\right)+X\left(s\right)p_{\mathrm{xy}}}{p_{\mathrm{zy}}}$$

From the same figure, *Z*(*s*) can be expressed as:

(6) $$Z\left(s\right)=-E\left(s\right)G_{Y}\left(s\right)p_{\mathrm{yz}}G_{Z}\left(s\right)+D\left(s\right)G_{Z}\left(s\right)$$

Substituting Eq. 6 for *Z*(*s*) into Eq. 5, we obtain:

(7) $$\begin{array}{ll} \frac{E\left(s\right)+X\left(s\right)p_{\mathrm{xy}}}{p_{\mathrm{zy}}}= & -E\left(s\right)G_{Y}(s)p_{\mathrm{yz}}G_{Z}(s)+D(s)G_{Z}(s)\\ \to E\left(s\right)= & \frac{-p_{\mathrm{xy}}}{1+p_{\mathrm{yz}}p_{\mathrm{zy}}G_{Y}\left(s\right)G_{Z}\left(s\right)}X(s)\\ & +\frac{p_{\mathrm{zy}}G_{Z}\left(s\right)}{1+p_{\mathrm{yz}}p_{\mathrm{zy}}G_{Y}\left(s\right)G_{Z}\left(s\right)}D(s) \end{array}$$

The second term in Eq. 7 represents the contribution to *E(s)* from *D*(*s*), the Laplace transform of the disturbance signal. We denote this term as *E*_*D*_(*s*) and its corresponding time domain function as *e*_*D*_(*t*):

(8) $$E_{D}\left(s\right)=L\left\{e_{D}\left(t\right)\right\}=\frac{p_{\mathrm{zy}}G_{Z}\left(s\right)}{1+p_{\mathrm{zy}}G_{Y}\left(s\right)p_{\mathrm{yz}}G_{Z}\left(s\right)}D\left(s\right)$$

Using the final value theorem and assuming a step disturbance (*D*(s) = 1/s), we can determine the steady-state error due to the disturbance as follows:

(9) $$\begin{array}{c} \lim_{t\to \infty }e_{D}\left(t\right)=\lim_{s\to 0}sE_{D}(s)=\lim_{s\to 0}\frac{sp_{\mathrm{zy}}G_{Z}\left(s\right)}{1+p_{\mathrm{zy}}G_{Y}\left(s\right)p_{\mathrm{yz}}G_{Z}\left(s\right)}D(s)\\ =\frac{p_{\mathrm{zy}}p_{y}}{p_{y}p_{z}+p_{\mathrm{yz}}p_{\mathrm{zy}}} \end{array}$$

According to Eq. 9, increasing either *p*_*yz*_ or *p*_*z*_ will reduce the steady-state error, because both terms are only in the denominator. Therefore, either a stronger negative feedback through the Mdm2 suppression of p53 (*p*_*yz*_) or less stable p53 (*p*_*z*_) can reduce the steady-state error. Interestingly, as stated previously, post-translational degradation of p53 by Mdm2 increases both *p*_*yz*_ and *p*_*z*_ (degradation rate), while a hypothetical transcriptional suppression would only increase *p*_*yz*_. Hence post-translational suppression of p53 may be more efficient at reducing steady-state error than transcriptional suppression, even though it is less energy efficient.

### Trade-off between steady-state and transient errors

The feedback has to minimize its transient response to disturbances in addition to steady-state error; otherwise, a temporary overshoot of the p53 level may trigger unintended effects. We therefore examine the percentage overshoot (%OS), which is the amount that the p53 level transiently overshoots the final steady-state level (expressed as a percentage of the final value). Because %OS is a function of the damping ratio (*ζ*), we derive a second-order transfer function representative of the block diagram in Figure 2A to determine its damping ratio (*ζ*). Substituting Eq. 5 for *E*(*s*) in Eq. 6, the transfer function *G*(*s*), which directly relates the input *X*(*s*) to the output *Z*(*s*), can be expressed as:

(10) $$\begin{array}{ll} Z\left(s\right) & =\left\{Z\left(s\right)p_{\mathrm{zy}}-X\left(s\right)p_{\mathrm{xy}}\right\}G_{Y}(s)p_{\mathrm{yz}}G_{Z}(s)+D(s)G_{Z}(s)\\ \to Z\left(s\right) & =\frac{p_{\mathrm{xy}}G_{Y}\left(s\right)p_{\mathrm{yz}}G_{Z}\left(s\right)X\left(s\right)}{1+p_{\mathrm{zy}}G_{Y}\left(s\right)p_{\mathrm{yz}}G_{Z}\left(s\right)}+\frac{D\left(s\right)G_{Z}\left(s\right)}{1+p_{\mathrm{zy}}G_{Y}\left(s\right)p_{\mathrm{yz}}G_{Z}\left(s\right)}\\ \to G\left(s\right) & =\frac{Z\left(s\right)}{X\left(s\right)}=\frac{p_{\mathrm{xy}}G_{Y}\left(s\right)p_{\mathrm{yz}}G_{Z}\left(s\right)}{1+p_{\mathrm{zy}}G_{Y}\left(s\right)p_{\mathrm{yz}}G_{Z}\left(s\right)}\\ & =\frac{p_{\mathrm{xy}}p_{\mathrm{yz}}}{s^{2}+\left(p_{y}+p_{z}\right)s+p_{y}p_{z}+p_{\mathrm{yz}}p_{\mathrm{zy}}} \end{array}$$

From Eq. 10, the natural frequency and damping ratio can be expressed as [24]:

(11) $$\omega_{n}\left(\mathrm{natural}\;\mathrm{frequency}\right)=\sqrt{p_{y}p_{z}+p_{\mathrm{yz}}p_{\mathrm{zy}}}$$

(12) $$\zeta \left(d\mathrm{amping}\;\mathrm{ratio}\right)=\frac{p_{y}+p_{z}}{2\sqrt{p_{y}p_{z}+p_{\mathrm{yz}}p_{\mathrm{zy}}}}$$

And the %OS is given by [7]:

(13) $$\;\mathrm{\%OS}=100\cdot e^{\left(\frac{-\varsigma \pi }{\sqrt{1-\varsigma^{2}}}\right)}\;$$

The typical time responses (unit step responses) for a second-order system with different damping ratios are shown in Figure 2B (*p*_*z*_ = *p*_*y*_ = 0.01 min^-1^). The %OS, or overshoot of the p53 level, decreases when the damping ratio increases (Figure 2C).

According to Eq. 12, increasing *p*_*yz*_ monotonically reduces the damping ratio and increases %OS, for *p*_*yz*_ only appears in the denominator. Since, transcriptional suppression can only influence *p*_*yz*_, a transcriptional negative feedback is limited by the trade-off between steady-state error and transient overshoot – increasing the strength of the negative feedback reduces steady-state error at the expense of increasing transient overshoot.

On the other hand, how does *p*_*z*_ affect the overshoot? If we take the partial derivative of the damping ratio in regard to *p*_*z*_, we get:

(14) $$\frac{\partial \zeta }{\partial p_{z}}=\frac{\sqrt{p_{y}p_{z}+p_{\mathrm{yz}}p_{\mathrm{zy}}}-\frac{p_{y}\left(p_{y}+p_{z}\right)}{2\sqrt{p_{y}p_{z}+p_{\mathrm{yz}}p_{\mathrm{zy}}}}}{2\left(p_{y}p_{z}+p_{\mathrm{yz}}p_{\mathrm{zy}}\right)}$$

(15) $$\;\frac{\partial \zeta }{\partial p_{z}}=0\Rightarrow p_{z}=p_{y}-\frac{2p_{\mathrm{yz}}p_{\mathrm{zy}}}{p_{y}}$$

The second-order partial derivative of Eq. 14 shows that *ζ* reaches the minimum value at $p_{z}=p_{y}-\frac{2p_{\mathrm{yz}}p_{\mathrm{zy}}}{p_{y}}\left(p_{y},p_{z}>0\to \overset{2}{\underset{y}{p}}>2p_{\text{zy}}p_{\text{zy}}\right)$. When $p_{z}<p_{y}-\frac{2p_{\mathrm{yz}}p_{\mathrm{zy}}}{p_{y}}$, *ζ* decreases with *p*_*z*_; when $p_{z}>p_{y}-\frac{2p_{\mathrm{yz}}p_{\mathrm{zy}}}{p_{y}}$, *ζ* increases with p_z_. This suggests that when $p_{z}>p_{y}-\frac{2p_{\mathrm{yz}}p_{\mathrm{zy}}}{p_{y}}$, increasing p_z_ can decrease both the steady-state error and overshoot (which is inverse to the damping ratio). This insight potentially explains why cells choose to spend extra energy producing and then actively degrading p53 under normal conditions – it rejects disturbances by reducing both the steady-state error and the transient overshoot. Furthermore, post-translational suppression influences both *p*_*yz*_ and *p*_*z*_, because the Mdm2-mediated degradation rate of p53 depends on both Mdm2 and p53 concentrations. This suggests that post-translational suppression can achieve a better trade-off than transcriptional suppression, which can only influence *p*_*yz*_.

**Figure 3:**
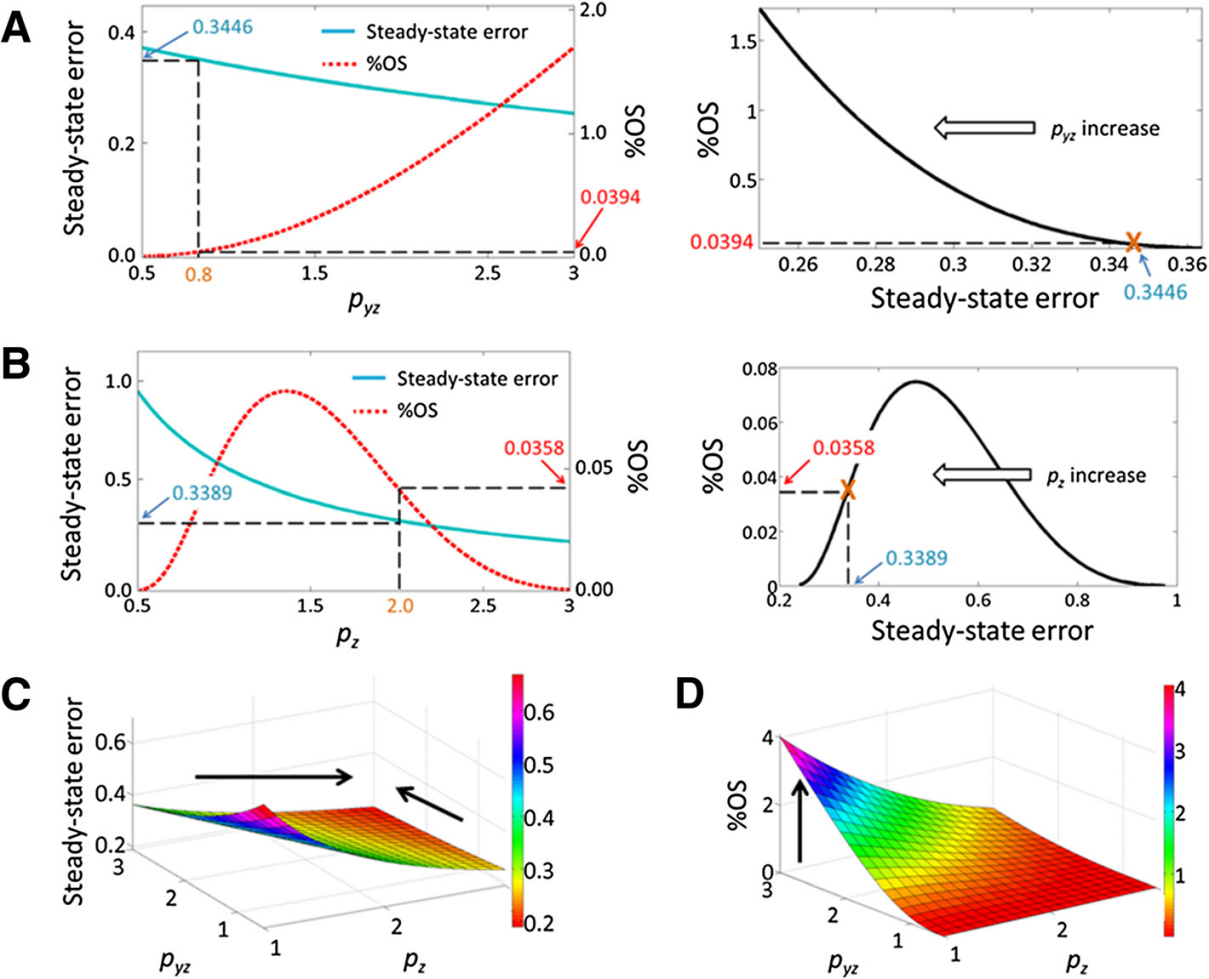
**Trade-off between steady-state error and transient overshoot. (A)** Steady-state error and %OS (left panel) and the trade-off curve (right panel) when *p*_*yz*_ is increased from 0.5 to 3.0. The orange cross denotes the operating point (*p*_*yz*_ = 0.8). **(B)** Steady-state error and %OS (left panel) and the trade-off curve (right panel) when *p*_*z*_ is increased from 0.5 to 3.0. The orange cross denotes the operating point (*p*_*z*_ = 2.0). **(C)** Increasing *p*_*yz*_ or *p*_*z*_ can reduce the steady-state error. **(D)** Increasing *p*_*yz*_ can increase %OS. A better operating point can be achieved by simultaneously increasing *p*_*z*_.

To quantitatively verify these analytical insights, we calculated the steady-state error, damping ratio, and %OS using published, experimentally measured parameter values (*p*_*yz*_ = 0.8 h^-1^[23], *p*_*zy*_ = 0.8 h^-1^[23], *p*_*y*_ = 2.0 h^-1^ (half-life ≈ 20 min) [25], *p*_*z*_ = 2.0 h^-1^ (half-life ≈ 20 min) [26]). As *p*_*yz*_ increases from 0.5 to 3.0, the steady-state error decreases while the %OS increases (the damping ratio decreases) (Figure 3A, left panel). Hence there is a trade-off between steady-state error and transient overshoot (Figure 3A, right panel), consistent with the previous analysis. Any further decrease of the steady-state error from the system operating point (*p*_*yz*_ = 0.8) will have to pay a hefty penalty for transient overshoot. On the other hand, as *p*_*z*_ is increased from 0.5 to 3.0, the %OS initially increases but then decreases (Figure 3B, left panel), exactly as Eqs. 14 and 15 have indicated. The system operates at a point (*p*_*y*_ = 2.0, *p*_*z*_ = 2.0) where $p_{z}\geq p_{y}-\frac{2p_{\mathrm{yz}}p_{\mathrm{zy}}}{p_{y}}$, which allows the system to reduce both the steady-state error and %OS through *p*_*z*_ (Figure 3B, right panel). The combined effect of *p*_*yz*_ and *p*_*z*_ is shown in Figure 3C and D. The steady-state error can be decreased by either increasing *p*_*yz*_ or *p*_*z*_ (Figure 3C), but increasing *p*_*yz*_ can increase %OS (Figure 3D), as shown by the arrows in the figures. Therefore, by modulating both *p*_*yz*_ and *p*_*z*_, post-translational suppression can achieve better steady-state error and %OS than transcriptional suppression, which only modulates *p*_*yz*_.

Interestingly, the fact that *p*_*z*_ can improve both steady-state error and %OS seems to suggest that cells should keep increasing *p*_*z*_ to achieve ever better robustness. However, it is worth noting that there is another trade-off factor that ultimately comes into the picture, which is the energy cost. Higher *p*_*z*_ means that the cells have to spend more resources to rapidly produce and then degrade p53 in a “futile” cycle, so the degradation rate cannot be increased indefinitely. The cell has to balance between robustness and energy spending to achieve the optimal operating point.

Altogether, our analytical and quantitative analyses suggest that cells spend extra energy to produce and degrade p53 simultaneously under normal conditions in order to maintain a more robust p53 level. By modulating both *p*_*yz*_ and *p*_*z*_, post-translational suppression of p53 enables the p53-Mdm2 feedback to achieve a better trade-off by reducing both steady-state errors and transient overshoots. On the contrary, transcriptional suppression only modulates *p*_*yz*_, which does not improve the overall trade-off.

### Robustness analyses with pole plots

As mentioned at the beginning of the paper, gene networks fluctuate over time under the influence of extrinsic and intrinsic noise. The range of *p*_*yz*_ and *p*_*z*_ in Figure 3 demonstrated the robustness trade-off between steady-state and transient disturbance rejection, but it will be useful to examine the trade-off within the range of parameter fluctuation that a realistic cell has to experience. To estimate the time-varying parameter values from time-series data of the p53-Mdm2 network [21], we constructed a corresponding discrete-time model, which can be described by Eqs. 16–18:

(16) $$x\left(i\right)=x\left(i-1\right)$$

(17) $$y\left(i\right)=w_{\mathrm{zy}}z\left(i-1\right)-w_{\mathrm{xy}}x\left(i-1\right)+w_{y}y\left(i-1\right)$$

(18) $$z\left(i\right)=-w_{\mathrm{yz}}y\left(i-1\right)+w_{z}z\left(i-1\right)$$

where x(*i*), y(*i*), and z(*i*) represent ATM, Mdm2, and p53 levels respectively. As before, the ATM level is assumed to be low and constant under normal conditions. *w*_*xy*_ represents the suppression of Mdm2 by ATM*, w*_*yz*_ represents the suppression of p53 by Mdm2, *w*_*zy*_ represents the transcriptional activation of Mdm2 by p53, *w*_*y*_ represents the stability of Mdm2 (1-*w*_*y*_ represents Mdm2 degradation), and *w*_*z*_ represents the stability of p53 (1-*w*_*z*_ represents p53 degradation). Note that *w*_*y*_ and *w*_*z*_ represent stability rather than degradation, opposite to *p*_*y*_ and *p*_*z*_. Block diagram representation of the discrete-time feedback model is shown in Figure 2D. Consistent with the continuous model, the discrete-time model confirms that the steady-state error can be decreased by increasing *w*_*yz*_ or decreasing *w*_*z*_ (Eq. 19, also see Eq. S11 in Additional file 1). Note that decreasing *w*_*z*_ means increasing p53 degradation and corresponds to increasing *p*_*yz*_.

(19) $$\lim_{i\to \infty }e_{D}\left(i\right)==\frac{w_{\mathrm{zy}}\left(1-w_{y}\right)}{\left(1-w_{y}\right)\left(1-w_{z}\right)+w_{\mathrm{yz}}w_{\mathrm{zy}}}$$

**Figure 4:**
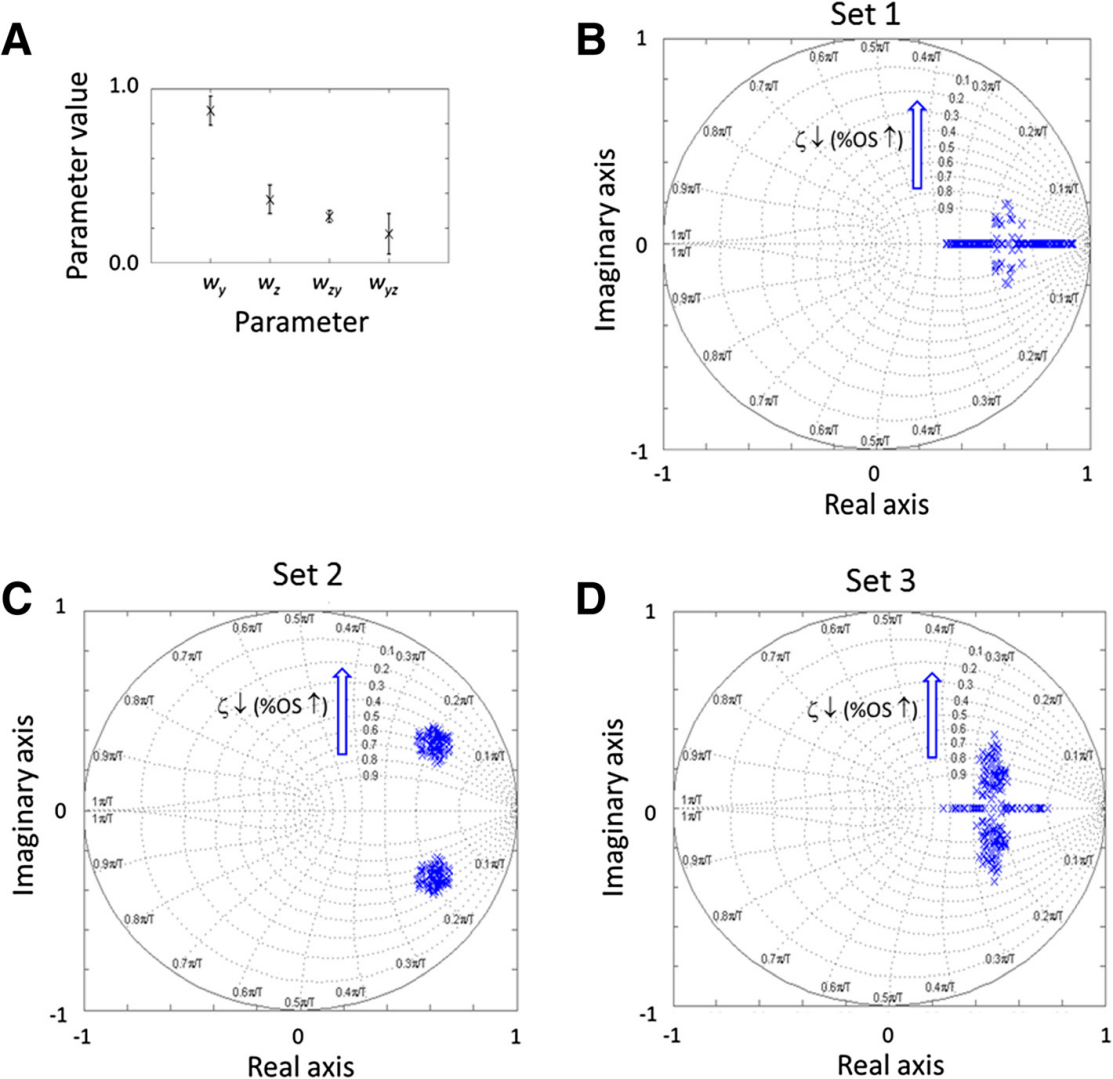
**Stability analysis. (A)** Mean and standard deviation of each parameter identified by the adaptive filter algorithm: *w*_*y*_: 0.8737±0.0830, *w*_*z*_: 0.3627±0.0825, *w*_*zy*_: 0.2662±0.0343, *w*_*yz*_: 0.1656±0.1169. **(B)** Pole map under the DNA damage condition when the feedback is weakened. The poles are close to the real axis and the corresponding damping ratios range mostly from 0.8 to 1.0. **(C)** Poles are located higher with bigger imaginary components and lower damping ratios (0.4 – 0.8) when *w*_*yz*_ is increased. **(D)** Poles are closer to the real axis with bigger damping ratios compared to **(C)** when *w*_*z*_ is decreased proportionally. *T* in the figure denotes the sampling period (0.11 hr). The numbers next to the arrow indicate the corresponding damping ratios.

We used the time-series experimental data [21] and an NLMS adaptive filter [27,28] to track the time-varying parameters of the p53-Mdm2 model (see Additional file 1) (Figure 4A). The published experimental data [21] and MATLAB code used to estimate the parameter ranges are included in Additional files 2, 3, 4 and 5. Based on the estimates, we examined how *w*_*yz*_ and *w*_*z*_ affect %OS.

Using Z-transform [29], we first derived a transfer function, *G*(*z*), which represents the feedback system shown as the block diagram in Figure 2D (see Additional file 1):

(20) $$\begin{array}{ll} G\left(z\right) & =\frac{Z\left(z\right)}{X\left(z\right)}=\frac{w_{\mathrm{xy}}G_{Y}\left(z\right)w_{\mathrm{yz}}G_{Z}\left(z\right)}{1+w_{\mathrm{zy}}G_{Y}\left(z\right)w_{\mathrm{yz}}G_{Z}\left(z\right)}\\ & =\frac{w_{\mathrm{xy}}w_{\mathrm{yz}}}{z^{2}-\left(w_{y}+w_{z}\right)z+w_{y}w_{z}+w_{\mathrm{yz}}w_{\mathrm{zy}}} \end{array}$$

To investigate the transient behavior and stability of G(z), we plotted its poles using the MATLAB Robust Control Toolbox (Figure 4B-D), which can be reproduced using the supplementary MATLAB file (Additional file 6). For each map, 100 points were calculated based on Monte Carlo sampling of the estimated parameter range in Figure 4A. We first plotted the poles for the DNA damage condition, under which the Mdm2 suppression of p53 (the negative feedback) is weakened to stop disturbance rejection (Figure 4B). Consistent with the weakening of the feedback, the plot shows that the poles are mostly real and the system is stable, with damping ratios ranging from 0.8 to 1.0.

Western blot measurements of the total and ubiquitinated p53 levels showed that the suppression of p53 is 3.67 fold higher under the normal condition compared to the DNA damage condition [30]. We first increased *w*_*yz*_ by 3.67 fold (from 0.1656±0.1169 to 0.6708±0.1169) to evaluate how transcriptional suppression will affect transient overshoot and stability. The increased *w*_*yz*_ values shifted poles higher on the map compared to the DNA damage condition (Figure 4C). The bigger imaginary components of the poles indicate that the damping ratios are lower (0.4 – 0.8) and the corresponding %OS values are higher for the system, therefore confirming our previous conclusion that transcriptional suppression reduces the steady-state error at the expense of transient overshoot and stability.

However, if the increase of *w*_*yz*_ is accompanied by a decrease of *w*_*z*_ (stability of p53) by 3.67 fold (0.3627±0.0825 to 0.0998±0.0825), which approximates post-translational suppression, the poles are located closer to the real axis. The smaller imaginary components of the poles indicate that the damping ratios are greater (the %OS values are smaller). Therefore, post-translational suppression can reduce the steady-state error without a hefty penalty of transient overshoot (%OS) and stability, unlike transcriptional suppression.

### Post-translational regulation enables faster responses with predictable level shifts

**Figure 5:**
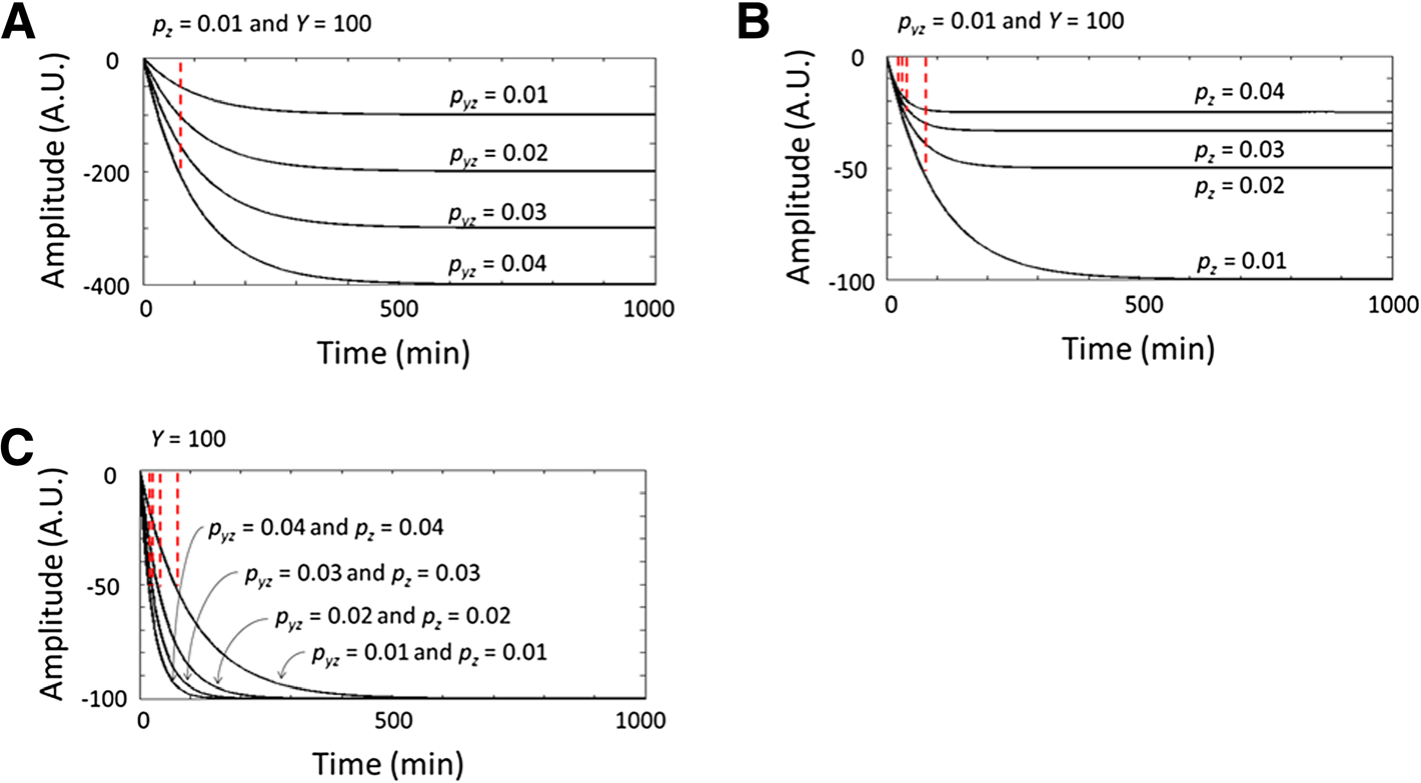
**Step responses of p53. (A)***p*_*yz*_ does not influence the response time. **(B)***p*_*z*_ decreases the response time but also the amplitude of the response. **(C)** Increasing *p*_*yz*_ while decreasing *p*_*z*_ enables faster response time with constant amplitude.

Post-translational regulation has another benefit over transcriptional regulation – it enables faster responses with more consistent amplitude. This is biologically significant, as cells must react quickly to external modulators (e.g. ATM) and stress conditions with a predictable shift of p53 levels. From Eq. 3, the step response of the p53-Mdm2 feedback can be shown as

(21) $$\begin{array}{l} \;\\ z\left(t\right)=\frac{‒p_{\mathrm{yz}}\cdot Y}{p_{z}}\left(1-e^{-p_{z}t}\right) \end{array}$$

with the assumption that *y*(t) has a constant value of *Y*. Eq. 21 suggests that *p*_*z*_ but not *p*_*yz*_ determines the response time (the time needed for *z*(*t*) to reach the half steady-state value) (Figure 5A and B). However, the increase in speed comes at a cost; it decreases the response amplitude as shown in Figure 5B. Interestingly, increasing both *p*_*yz*_ and *p*_*z*_ allows the system to achieve a faster response time with constant steady-state amplitude (Figure 5C), because the respective increases of *p*_*yz*_ and *p*_*z*_ offset each other at the steady level (Eq. 21). Therefore, by modulating both *p*_*yz*_ and *p*_*z*_, post-translational suppression can generate more prompt and consistent p53 response to stress conditions. Indeed, cells respond to DNA damage by modulating the post-translational degradation of p53.

### Validation by a non-linear, mechanistic model

So far, our analyses have been performed using the linear p53-MDM2 feedback model (Eq. 1–3) modified from Geva-Zatorsky et al. [23]. Even though this model matches experimental measurements [23], it does not capture the non-linear aspects of the negative feedback loop. Therefore, the linear model, and hereby its analyses, can only be applied to the first order approximation.

To test whether our findings are still valid when non-linear effects are taken into consideration, we built a mechanistic model of the p53-MDM2 feedback [Eqs. 22–25]:

(22) $$\frac{d\left[\mathrm{Mdm}2_{\mathrm{mRNA}}\right]}{\mathrm{dt}}=\beta_{1}\frac{{\left[P53\right]}^{n}}{K_{1}{}^{n}+{\left[P53\right]}^{n}}-\alpha_{1}\left[\mathrm{mdm}2_{\mathrm{mRNA}}\right]$$

(23) $$\frac{d\left[\mathrm{Mdm}2\right]}{\mathrm{dt}}=\beta_{2}\left[\mathrm{mdm}2_{\mathrm{mRNA}}\right]-\alpha_{2}\left[\mathrm{Mdm}2\right]$$

(24) $$\frac{d\left[p53_{\mathrm{mRNA}}\right]}{\mathrm{dt}}=\beta_{3}-\alpha_{3}\left[p53_{\mathrm{mRNA}}\right]$$

(25) $$\frac{d\left[P53\right]}{\mathrm{dt}}=\beta_{4}\left[p53_{\mathrm{mRNA}}\right]-\alpha_{4}\left[P53\right]-\gamma \frac{\left[P53\right]}{K_{2}+\left[P53\right]}\left[\mathrm{Mdm}2\right]$$

where, [*mdm2*_*mRNA*_], [*Mdm2*], [*p53*_*mRNA*_], and [*P53*] denote the mRNA and protein levels of p53 and Mdm2. β_1_ and β_3_ denotes transcription rates, and β_2_ and β_4_ denote translation rates of Mdm2 and p53. *α*_*s*_ (s = 1,2,3,4) denote mRNA and protein degradation rates. Transcriptional activation of Mdm2 by p53 is modeled by a Hill function, where *n* is the hill coefficient and *K*_1_ is the dissociation constant. Mdm2-mediated p53 ubiquitination and degradation are modeled by a Michaelis-Menten (MM) function as described by Xu et al. [31]. *γ* is the reaction rate of p53 ubiquitination by Mdm2, and *K*_2_ is the saturation constant.

**Figure 6:**
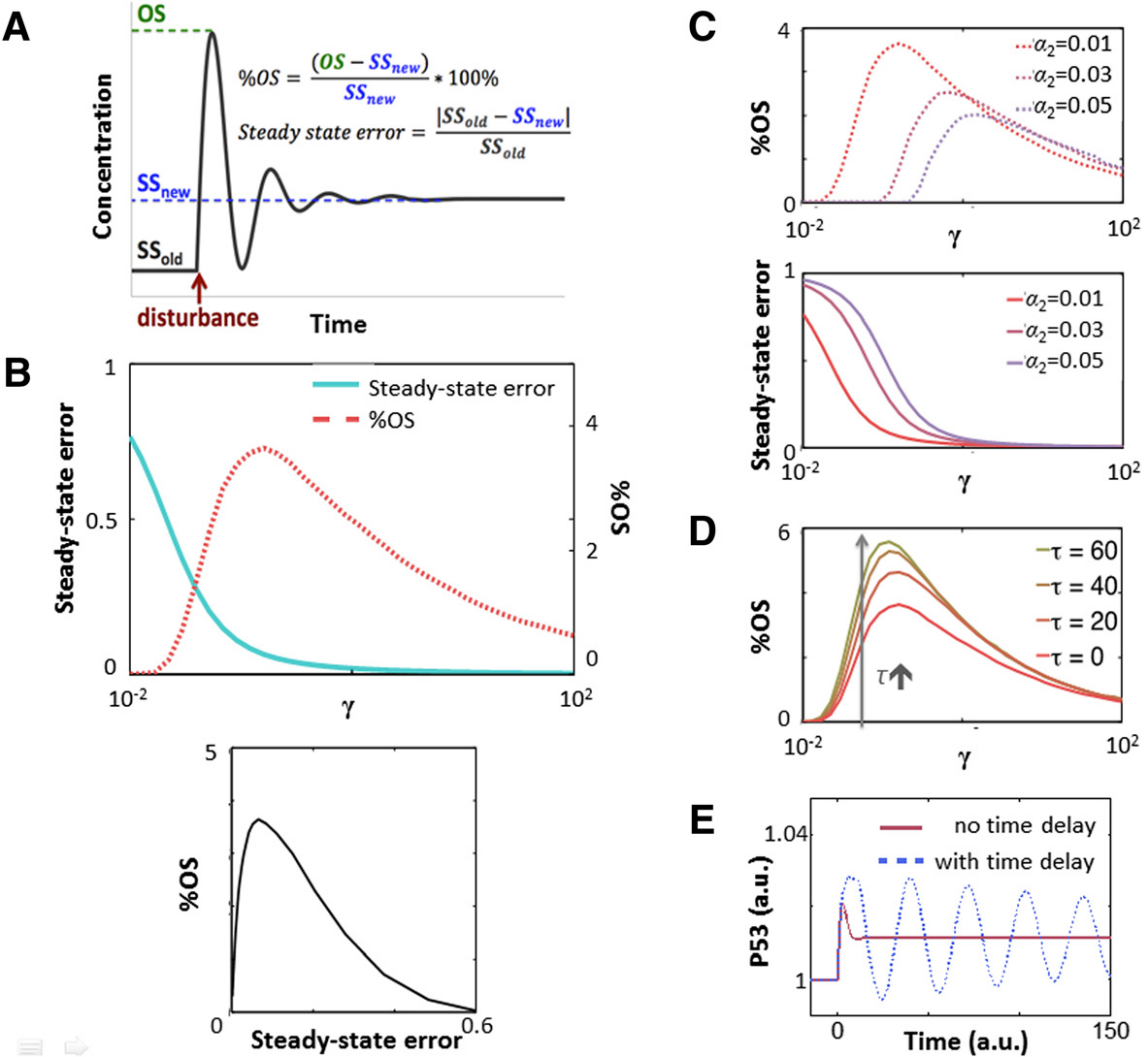
**Disturbance rejection simulation of a mechanistic model. (A)** Simulation of a step function disturbance to p53 production. The equations illustrate how the steady-state error and %OS are calculated. **(B)** Steady-state error and %OS (top panel) and the trade-off curve (bottom panel) when *γ* increases. **(C)** Plots of %OS and steady-state error vs. *γ* with varying Mdm2 levels. **(D)** Plots of %OS vs. *γ* with varying time delay. **(E)** Time delay can cause sustained oscillation (*β*_*1*_=1, *β*_*2*_=1, *β*_*3*_=1, *β*_*4*_=1, *α*_*1*_ =1, *α*_*2*_ =0.01, *α*_*3*_ =1, *α*_*4*_ =0.01, *n* =2, *K*_*1*_ =10, *K*_*2*_ =100, *γ=10*^*-2*^*- 10*^*2*^).

To measure disturbance rejection, p53 production was increased by 1% from its steady-state level (simulated by a step function). Steady-state error and %OS were then calculated based on the following equations:

(26) $$\begin{array}{cc} \mathrm{Steady}-\mathrm{state} & \mathrm{error} \end{array}=\frac{\left|SS_{\mathrm{old}}-SS_{\mathrm{new}}\right|}{SS_{\mathrm{old}}}$$

(27) $$\mathrm{\%OS}=\frac{\left|\mathrm{OS}-SS_{\mathrm{new}}\right|}{SS_{\mathrm{new}}}$$

where OS is the overshoot level, SS_old_ is the p53 steady-state level before perturbation, and SS_new_ is the p53 steady-state level after perturbation (Figure 6A).

When *γ*, the degradation rate of p53 by Mdm2-mediated ubiquitination, increases, the %OS initially increases but then decreases, which allows the system to reduce both the steady-state error and %OS through gamma (Figure 6B). This plot is similar to the previous analysis with the linear model when p_z_ is increased (Figure 3B). Therefore, the mechanistic model confirmed the insight from the linear model that post-translational suppression enables the p53-Mdm2 negative feedback to reduce both steady-state error and %OS.

We explored the parameter space to examine the different operating regions of the non-linear model, and found that the above tradeoff trend is generally preserved even though the absolute values of steady-state error and %OS vary (Figure 6C). This is probably due to the fact that the model is roughly piece-wise linear at each operating point when given a modest disturbance, so that higher-order effects do not dominate the first-order behavior predicted by the linear model.

Besides non-linearity, the linear model is also overly simplistic in terms of another critical aspect of the p53-Mdm2negative feedback, which is time delay. Time delay in a negative feedback loop can decrease stability, increase %OS and cause sustained oscillation. To examine how time delay affects disturbance rejection, we added explicit delay terms between transcription and translation [Eqs. 28–31]:

(28) $$\frac{d\left[\mathrm{mdm}2_{\mathrm{mRNA}}\right]}{\mathrm{dt}}=\beta_{1}\frac{{\left[P53\right]}^{n}}{K_{1}{}^{n}+{\left[P53\right]}^{n}}-\alpha_{1}\left[\mathrm{mdm}2_{\mathrm{mRNA}}\right]$$

(29) $$\frac{d\left[\mathrm{Mdm}2\right]}{\mathrm{dt}}=\beta_{2}\left[\mathrm{mdm}2_{\mathrm{mRNA}}\right]\left(t-\tau \right)-\alpha_{2}\left[\mathrm{Mdm}2\right]$$

(30) $$\frac{d\left[p53_{\mathrm{mRNA}}\right]}{\mathrm{dt}}=\beta_{3}-\alpha_{3}\left[p53_{\mathrm{mRNA}}\right]$$

(31) $$\begin{array}{ll} \frac{d\left[P53\right]}{\mathrm{dt}}= & \beta_{4}\left[p53_{\mathrm{mRNA}}\right]\\ & \times \left(t-\tau \right)-\alpha_{4}\left[P53\right]-\gamma \frac{\left[P53\right]}{K_{2}+\left[P53\right]}\left[\mathrm{Mdm}2\right] \end{array}$$

where *τ* denotes the time delay between transcription and translation.

Simulations with varying time delay reveal that delay does not affect steady-state error but increases %OS, which is consistent with the proposition that time delay decreases stability of negative feedback loops (Figure 6D). A longer delay also increases the likelihood of sustained oscillation (Figure 6E).

### Stochastic simulation

**Figure 7:**
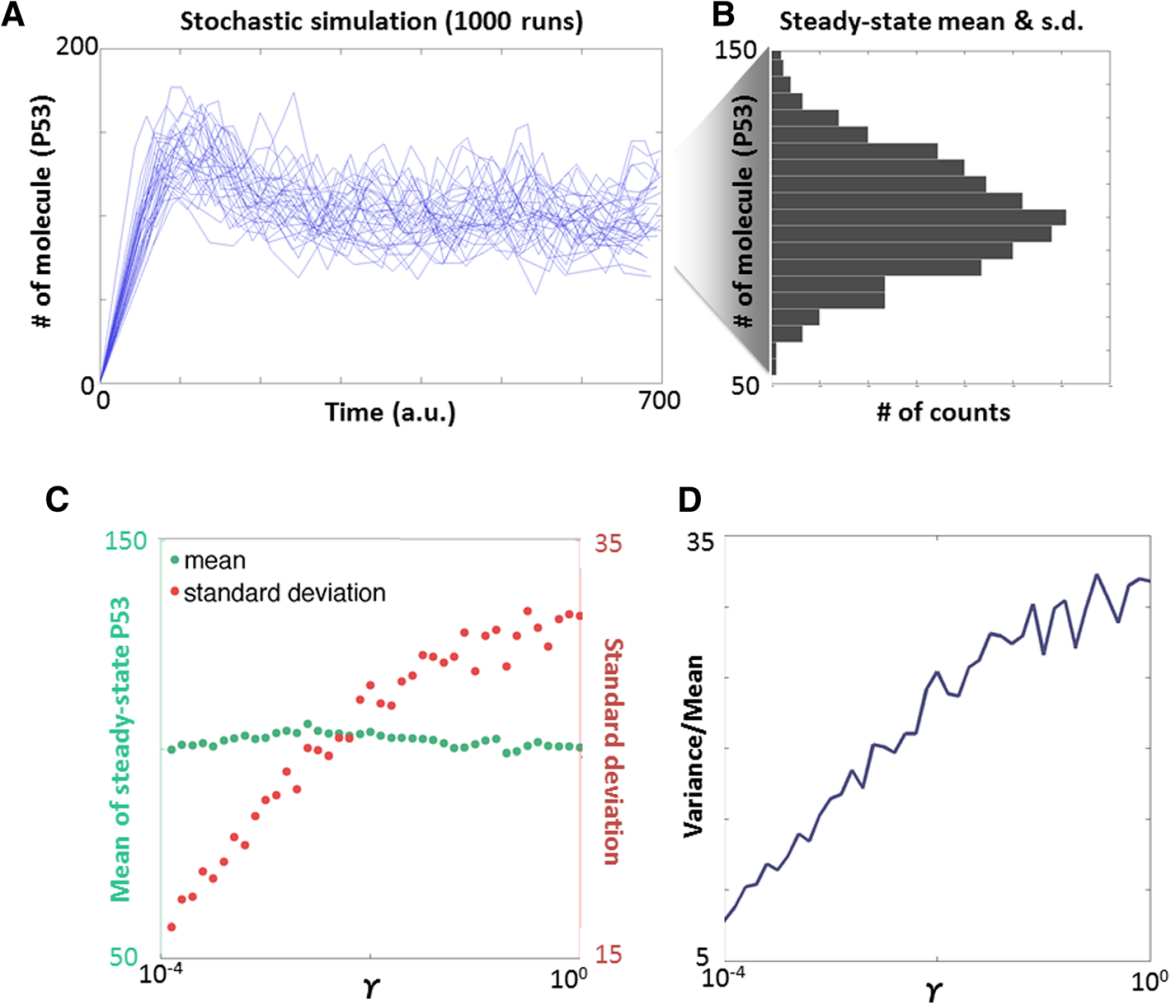
**Stochastic simulation of the feedback loop. (A)** p53 levels during repeated stochastic runs. **(B)** Mean and standard deviation of the steady-state p53 levels calculated from stochastic runs. **(C)** Mean and standard deviation of p53 with varying *γ*. We maintained a relatively constant p53 level by adjusting its natural degradation rate accordingly. **(D)** Variance normalized by mean increases with *γ*.

So far, the deterministic p53-Mdm2 models have shown how the negative feedback helps reject external disturbances. However, how does the feedback affect intrinsic, stochastic noise? To understand this issue, we constructed a mechanistic, kinetic model and performed stochastic simulations using the MATLAB SimBiology Toolbox (see Additional file 1) (Figure 7A). The mean and standard deviation of the steady-state p53 levels are calculated based on stochastic runs (Figure 7B). We varied *γ*, the degradation rate of Mdm2-ubiquitinated p53, while keeping the p53 level constant, to investigate how the negative feedback affects p53 variance (Figure 7C). The stochastic noise was then calculated as the variance normalized by the mean (Figure 7D). These stochastic simulations reveal that stochastic noise on p53 increases when *γ* increases. This suggests that while Mdm2-mediated p53 degradation improves disturbance rejection, it pays the price of amplifying stochastic noise. Hence, the p53-Mdm2 negative feedback loop has to balance the trade-off between disturbance rejection and stochastic noise.

## Conclusions

In summary, our analyses indicate that cells maintain robust p53 levels and reject disturbances by simultaneously producing and degrading p53, even though this process is more energy intensive. Within the p53-Mdm2 negative feedback loop, post-translational suppression of p53 by Mdm2 achieves a better trade-off between steady-state and transient errors than transcriptional suppression, which potentially explains why the former has been experimentally observed in the cell. Furthermore, post-translational suppression enables p53 to respond faster to stress conditions with a more predictable level shift. Understanding these nuances allows us to appreciate the complexity of regulatory networks, which will potentially lead to better therapeutics.

Besides ubiquitination, p53 activity is also heavily regulated by phosphorylation and nucleocytoplasmic shuttling [32-35]. Interestingly, these post-translational processes provide similar beneficial tradeoff between steady-state errors and overshoot, while being faster and less energy intensive than protein degradation. It is probably not a coincidence then that p53 has multiple phosphorylation sites and is shuttled alongside Mdm2 between nucleus and cytoplasm. Therefore, protein degradation is only one of several post-translational mechanisms that enhance the robustness of the system.

Increasing degradation rather than reducing production might be a common strategy evolved by biological systems for robustness. For example, hematopoietic stem cells continuously go through apoptosis [36], which seems energy-inefficient and futile as the protein degradation we discussed. However, our analysis would suggest that regulating apoptosis rather than cell division may enable the stem cell population to become more robust to disturbances and respond faster to changes. The fact that biological systems employ mechanisms for robustness at many different levels raises an interesting question - how does robustness at each level contribute to the overall robustness of the whole system? Undoubtedly challenging, attempts to answer this question will help unravel the underlying design principles of complex biological systems.

## Methods

### Computational methods

Ordinary differential and difference equations were used for physics-based modeling of the p53-Mdm2 feedback loop (see Additional file 1 for the derivation of the p53-Mdm2 discrete-time model from underlying physics). Parameter ranges of the discrete-time model were estimated using the Normalized Least Mean Squares (NLMS) method detailed in Additional file 1. The experimental data [21] and MATLAB (Mathworks, USA) codes are provided as (Additional files 1, 2, 3, 4 and 5). Steady-state and transient error analysis was performed using Laplace and Z-domain analysis (see Additional file 1 for steady-state error analysis using the discrete-time model). The Monte Carlo method and MATLAB Robust Control Toolbox (Mathworks, USA) were used for the estimation-based robustness analysis (see Additional file 6).

### Image extraction and fluorescence quantification

285 Image frames were extracted from the video file [21] and the fluorescence quantification of p53 and Mdm2 was carried out using the LabVIEW Vision Assistant 2010 (National Instruments, USA). We manually marked the location of each cell nucleus in each frame and 285 data points were obtained for each protein.

## Competing interests

The authors declare that they have no competing interests.

## Authors’ contributions

YS conceived of and designed the study, performed linear, discrete-time and robustness analyses and drafted the manuscript. KC carried out non-linear and stochastic analyses. AHS assisted in the adaptive filter analysis. BH participated in designing the robustness analysis. XS conceived of the study, participated in its design, and helped to draft the manuscript. All authors have read and approved the manuscript.

## Supplementary Material

Additional file 1 Supplementary document. Click here for file

Additional file 2 p53 data. Click here for file

Additional file 3 Mdm2 data. Click here for file

Additional file 4 p53-Mdm2 parameter tracking using NLMS (Mdm2 estimation). Click here for file

Additional file 5 p53-Mdm2 parameter tracking using NLMS (p53 estimation). Click here for file

Additional file 6 p53-Mdm2 discrete uncertain model: stability analysis. Click here for file
