# Supplementary material for: Post-translational regulation enables robust p53 regulation
Source: BMC Syst Biol. 2013 Aug 30;7:83. doi: 10.1186/1752-0509-7-83 (PMC3844394; doi:10.1186/1752-0509-7-83)
Supplement: Additional file 1 — Supplementary document. [file 1752-0509-7-83-S1.doc]

**Supplementary document**

**Discrete-time model**

If gene *a* activates gene *b*, the protein *B* level can be expressed in a discrete-time model as:

$b(i)=p_{ab}a(i-1)+p_{b}b(i-1)$ [S1]

where *i* is the sampling index. *p_ab_* represents how the previous value*, a*(*i-*1)*,* affects the current value*, b*(*i*). *p_ab_* is a positive number and the sign in front of *p_ab_* indicates whether *a* activates or inhibits b. *p_b_* represents how the previous value*, b*(*i-*1)*,* affects its current value, b(*i*). The parameter *p_b_* represents the stability of the protein B (0 < *p_b_* < 1). *p_b_* should be less than one unless protein B activates gene *b* (positive autoregulation). As *p_b_* increases (closer to 1) *b*(*i*) becomes less different from *b*(*i*-1), indicating that protein B levels are more stable. Lower *p_b_* values (close to 0) correspond to higher protein degradation rates and (1-*p_b_*) corresponds to the protein degradation rate used in the continuous model (e.g. Eq. 2 or 3 in the main text). Using Z-transform, we can derive the transfer function of the system from Eq. S1:

$B(z)=p_{ab}A(z)z^{-1}+p_{b}B(z)z^{-1}\text{ or B(z) }=\text{ }\frac{p_{ab}A(z)}{z-p_{b}}$ [S2]

By letting *A’*(*z*) = *p_ab_A*(*z*), the transfer function *G_B_*(*z*) can be expressed as:

$G_{B}(z)=\text{ }\frac{B(z)}{A'(z)}=\frac{1}{z-p_{b}}$ [S3]

Similarly, a discrete-time model for the p53-MDM2 system can be expressed as:

$x(i)=x(i-1)$ [S4]

$y(i)=w_{zy}z(i-1)-w_{xy}x(i-1)+w_{y}y(i-1)$ [S5]

$z(i)=-w_{yz}y(i-1)+w_{z}z(i-1)$ [S6]

where x(*i*), y(*i*), and z(*i*) represent ATM, Mdm2, and p53 levels respectively. The ATM level is assumed to be low and constant (Eq.S4); *w_xy_* represents suppression of Mdm2 by ATM*, w_yz_* represents transcriptional suppression of p53 by Mdm2, *w_zy_* represents transcriptional activation of MDM2 by p53, *w_y_* represents degradation/dilution of Mdm2, and *w_z_* represents degradation/dilution of p53.

**Steady-state error analysis using the discrete-time model**

Eqs. S4-6 can be represented as a block diagram as shown in **Figure 2D** using the *z*-transform [1] . *X*(*z*), *Y*(*z*), and *Z*(*z*) are the z-transforms of *x*(*i*), *y*(*i*), and *z*(*i*), respectively. *G_Y_*(*z*) and *G_Z_*(*z*) are the transfer functions that represent the Mdm2 and p53 systems, as derived earlier in Eq. S3:

$G_{Y}(z)=\frac{1}{z-w_{y}},\text{ }G_{Z}(z)=\frac{1}{z-w_{z}}$ [S7]

The error *E*(*z*) can be expressed as:

$E(z)=Z(z)w_{zy}-X(z)w_{xy}\text{ }\to\text{ }Z(z)=\frac{E(z)+X(z)w_{xy}}{w_{zy}}$ [S8]

From the block diagram, *Z*(*z*) can also be written as:

$Z(z)=-E(z)G_{Y}(z)w_{yz}G_{Z}(z)+D(z)G_{Z}(z)$ [S9]

Substituting Eq. S8 for *Z*(*z*) into Eq. S9, we obtain:

$\begin{matrix} \frac{E(z)+X(z)w_{xy}}{w_{zy}}=-E(z)G_{Y}(z)w_{yz}G_{Z}(z)+D(z)G_{Z}(z) \\ \to E(z)=\frac{-w_{xy}}{1+w_{yz}w_{zy}G_{Y}(z)G_{Z}(z)}X(z) \\ +\frac{w_{zy}G_{Z}(z)}{1+w_{yz}w_{zy}G_{Y}(z)G_{Z}(z)}D(z) \end{matrix}$ [S10]

The second term in Eq. S10 represents the contribution to *E(z)* by *D*(*z*). We denote this term by *E_D_*(*z*) and its corresponding time domain sequence as *e_D_*(*i*). Using the final value theorem and assuming a step disturbance (*D*(z) = 1/(1-*z*^-1^)), we can determine the steady-state error due to the disturbance as follows:

$\begin{matrix} \lim_{i\to\infty} e_{D}(i)=\lim_{z\to1} (z-1)E_{D}(z) \\ =\lim_{z\to1} (z-1)\frac{w_{zy}G_{Z}(z)}{1+w_{yz}w_{zy}G_{Y}(z)G_{Z}(z)}D(z) \\ =\frac{w_{zy}(1-w_{y})}{(1-w_{y})(1-w_{z})+w_{yz}w_{zy}} \end{matrix}$ [S11]

Therefore the steady-state error is reduced when *w_yz_* is increased or w_z_ is decreased, consistent with the continuous-time model. Substituting Eq. S8 for *E*(*z*) into Eq. S9, the transfer function *G*(*z*) can be expressed as:

$\begin{matrix} Z(z)=-\left\{Z(z)w_{zy}-X(z)w_{xy}\right\}G_{Y}(z)w_{yz}G_{Z}(z)+D(z)G_{Z}(z) \\ \to Z(z)=\frac{w_{xy}G_{Y}(z)w_{yz}G_{Z}(z)X(z)}{1+w_{zy}G_{Y}(z)w_{yz}G_{Z}(z)}+\frac{D(z)G_{Z}(z)}{1+w_{zy}G_{Y}(z)w_{yz}G_{Z}(z)} \\ \to G(z)=\frac{Z(z)}{X(z)}=\frac{w_{xy}G_{Y}(z)w_{yz}G_{Z}(z)}{1+w_{zy}G_{Y}(z)w_{yz}G_{Z}(z)} \\ =\frac{w_{xy}w_{yz}}{z^{2}-(w_{y}+w_{z})z+w_{y}w_{z}+w_{yz}w_{zy}} \end{matrix}$ [S12]

This transfer function is used for analyzing the %OS (also the damping ratio) when the parameter ranges are estimated as shown next.

**Parameter estimation using the NLMS algorithm**

Before estimating the parameters for the DNA damage condition (**Figure 1C**, **right panel**), we modify Eqs. S4-6. We first remove *x* (ATM) and the parameter *w_xy_* from the equations since the experimental data for ATM are not available. We attempt to justify this removal using two arguments. First, Eqs. S11 and S12 indicate that they play no role in determining the steady-state error and damping ratio (thus %OS). Second, we already have oscillating Mdm2 data (**Figure 1B**) affected by oscillating ATM so we can say that ATM is indirectly incorporated into our new model via Mdm2 although it is not explicitly shown. Another significant modification is that we add a new term *β* (*i*-1) to Eq. 22, where *β*(*i*) represents the amount of p53 produced at each iteration. Although it is difficult to estimate exact *β*(*i*) values from the figure, since the p53 mRNA levels do not change much upon DNA damage we assume they are constant (i.e., *β*(*i*) =*β*(*i-1*)) as described earlier. The constant value can be approximated by observing the slope of the rising curves (assuming the p53 degradation by Mdm2 is blocked by ATM in those curve intervals) at **Figure 1B**, which is about 3 A.U. per iteration. The modified discrete-time model for the p53-MDM2 feedback loop is given by:

$y(i)=w_{zy}z(i-1)+w_{y}y(i-1)$ [S13]

$z(i)=\beta(i-1)-w_{yz}y(i-1)+w_{z}z(i-1)$ [S14]

For the parameter vector *w_1_*(*i*) = [*w_y_*, *w_zy_*]*^T^*(Eq. S13), we used data vector *ϕ_1_*(*i*) as the regression (input) data and *y*(*i*) as the reference (output) data. *ϕ_1_*(*i*) is given by:

$\phi_{1}(i)=\left[y(i-1),z(i-1)\right]^{T}$ [S15]

For the parameter vector *w_2_*(*i*) = [*w_z_*, *w_yz_*]*^T^* (Eq. S14), we used data vector *ϕ_2_*(*i*) as the regression (input) data and *z*(*i*) as the reference (output) data. *ϕ_2_*(*i*) can be expressed as:

$\phi_{2}(i)=\left[z(i-1),-y(i-1)\right]^{T}$ [S16]

Using the NLMS, *w*(*i*) is updated according to the following equation.

$w_{N}(i+1)=w_{N}(i)+\mu(i)e(i)\phi_{N}(i)$ (*N* = 1 for Eq. S13, *N* = 2 for Eq. S14) [S17]

where $i$is the iteration index and $\mu(i)$is the iteration step size at time $i$. The error *e*(*i*) is computed as:

$e(i)=y(i)-\phi_{1}(i)^{T}w_{1}(i)$ [S18]

$e(i)=z(i)-\left\{\phi_{2}(i)^{T}w_{2}(i)+\beta(i-1)\right\}$ [S19]

The self-adjustable step size $\mu(i)$is chosen as:

$\mu(i)=\frac{\mu}{\varepsilon+\left\|\phi{}_{N}(i)\right\|^{2}}$ [S20]

where$\mu$is the fixed iteration step size (0.5 was used for the parameter estimation) and $\varepsilon$in the denominator is a very small positive constant (1×10^-6^).

**Stochastic model**

Table 1: Reactions of P53-Mdm2 feedback loop.

| Number | Reaction | Description | Reaction rate |
| --- | --- | --- | --- |
| 1 | P53→mdm2_mRNA_ | Transcription of mdm2_mRNA_ activated by P53 | β_1_*P53 |
| 2 | mdm2_mRNA_ → *Φ* | Degradation of mdm2_mRNA_ | α_1_*mdm2_mRNA_ |
| 3 | mdm2_mRNA_→Mdm2 | Translation of Mdm2 | β_2*_mdm2_mRNA_ |
| 4 | Mdm2 → *Φ* | Degradation of Mdm2 | α_2_*Mdm2 |
| 5 | *Φ*→p53_mRNA_ | Transcription of p53_mRNA_ | β_3_ |
| 6 | p53_mRNA_ → *Φ* | Degradation of p53_mRNA_ | α_3_*p53_mRNA_ |
| 7 | p53_mRNA_ → P53 | Translation of P53 | β_4_*p53_mRNA_ |
| 8 | P53 → *Φ* | Basal degradation of P53 | α_4_*P53 |
| 9 | P53 $\xrightarrow{Mdm2}$ *Φ* | P53 ubiquitionation by Mdm2 | ϒ*P53*Mdm2 |

Reaction rate equations:

$$\frac{d[mdm2_{mRNA}]}{dt}=\beta_{1}[P53]-\alpha_{1}[mdm2_{mRNA}]$$

$$\frac{d[Mdm2]}{dt}=\beta_{2}[mdm2_{mRNA}]-\alpha_{2}[Mdm2]$$

$$\frac{d[p53_{mRNA}]}{dt}=\beta_{3}-\alpha_{3}[p53_{mRNA}]$$

$$\frac{d[P53]}{dt}=\beta_{4}[p53_{mRNA}]-\alpha_{4}[P53]-\gamma[P53][Mdm2]$$

Parameters:

β_1_ = 1 (1/time unit), β_2_ = 0.1 (1/time unit), β_3_ = 1 (# of molecule/time unit), β_4_ = various (1/time unit), α_1_ = 5 (1/time unit), α_2_ = 0.01 (1/time unit), α_3_ = 0.1 (1/time unit), α_4_ = 0.01 (1/time unit), ϒ = various (1/(# of molecule * time unit)
