# Supplementary material for: Post-translational regulation enables robust p53 regulation
Source: BMC Syst Biol. 2013 Aug 30;7:83. doi: 10.1186/1752-0509-7-83 (PMC3844394; doi:10.1186/1752-0509-7-83)
Supplement: Additional file 5 — p53-Mdm2 parameter tracking using NLMS (p53 estimation). [file 1752-0509-7-83-S5.doc]

% p53-Mdm2 parameter tracking using NLMS (p53 estimation)

% Yong-Jun Shin

% BioLab Cornell (2013)

%%

close all %close all figures

p53 = importdata ('Additional_file_2.txt'); % import p53 data

mdm2 = importdata ('Additional_file_3.txt'); % import mdm2 data

p53_basal = 10; % ATM-independent p53 production (p53 produced at every iteration)

%%

N = length(p53);

M=2; % 2: 1input+1output

mu = 0.5; % step-size for e-NLMS

epsilon = 1e-6;

w = zeros(M,N); % weight estimate

u = zeros(1,M); % regressor

for i=1:N-1

% p53 is estimated excluding the ATM-independent production effect.

u = [p53(i) -mdm2(i)];

p53_est(i) = u*w(:,i)+p53_basal;

e(i) = p53(i) - p53_est(i);

factor = epsilon + (norm(u)^2);

w(:,i+1) = w(:,i) + (mu/factor)*u'*e(i); % NLMS

end

figure

subplot(2,2,1)

plot(1:N,p53);

title('p53');

axis tight;

subplot(2,2,2)

plot(1:N-1,p53_est);

axis tight;

title('p53 est');

axis tight;

e_p53 = e;

subplot(2,2,3)

plot(1:N-1,e_p53);

axis tight;

title('Error signal');

axis tight;

w_z = w(1,:);

w_yz = w(2,:)

subplot(2,2,4)

plot(1:N,w_z,'r',1:N,w_yz,'b')%,1:N-1,w(2,1,:),1:N-1,w(3,1,:));

axis tight;

title('Wz (red), Wyz (blue)');

axis tight;
