# Supplementary material for: Post-translational regulation enables robust p53 regulation
Source: BMC Syst Biol. 2013 Aug 30;7:83. doi: 10.1186/1752-0509-7-83 (PMC3844394; doi:10.1186/1752-0509-7-83)
Supplement: Additional file 6 — p53-Mdm2 discrete uncertain model: stability analysis. [file 1752-0509-7-83-S6.doc]

% p53-Mdm2 discrete uncertain model: Stability analysis

% Yong-Jun Shin

% BioLab Cornell (2013)

% Requires MATLAB Robust Control Toolbox

%% Uncertain model Set 1

% y: Mdm2

% u: p53

clc

clear all;

close all;

mean_w_y = 0.8737;

std_w_y = 0.0830;

mean_w_z = 0.3627;

std_w_z = 0.0825;

mean_w_zy = 0.2662;

std_w_zy = 0.0343;

mean_w_yz = 0.1656;

std_w_yz = 0.1169;

u_w_y = ureal('u_w_y', mean_w_y, 'range', [mean_w_y-std_w_y mean_w_y+std_w_y]);

u_w_z = ureal('u_w_z', mean_w_y, 'range', [mean_w_z-std_w_z mean_w_z+std_w_z]);

u_w_zy = ureal('u_w_zy', mean_w_y, 'range', [mean_w_zy-std_w_zy mean_w_zy+std_w_zy]);

u_w_yz = ureal('u_w_yz', mean_w_y, 'range', [mean_w_yz-std_w_yz mean_w_yz+std_w_yz]);

w_xy = 0.1; % assume w_xy = 0.1

num = [w_xy*u_w_yz]; % numerator of G(z)

den = [1 -(u_w_y+u_w_z) u_w_y*u_w_z+u_w_yz*u_w_zy]; % denominator of G(z)

G = tf(num, den, 0.11); % discrete-time model with sampling period 0.11 hour

%Monte Carlo random sampling (100) of combinations of 4 uncertain parameters

%and the resulting pole-zero map

figure

pzmap(usample (G,100))

zgrid

%% Uncertain model Set 2

% y: Mdm2

% u: p53

clc

clear all;

close all;

mean_w_y = 0.8737;

std_w_y = 0.0830;

mean_w_z = 0.3627;

std_w_z = 0.0825;

mean_w_zy = 0.2662;

std_w_zy = 0.0343;

mean_w_yz = 0.6708; % 0.1656 --> 0.6708

std_w_yz = 0.1169;

u_w_y = ureal('u_w_y', mean_w_y, 'range', [mean_w_y-std_w_y mean_w_y+std_w_y]);

u_w_z = ureal('u_w_z', mean_w_y, 'range', [mean_w_z-std_w_z mean_w_z+std_w_z]);

u_w_zy = ureal('u_w_zy', mean_w_y, 'range', [mean_w_zy-std_w_zy mean_w_zy+std_w_zy]);

u_w_yz = ureal('u_w_yz', mean_w_y, 'range', [mean_w_yz-std_w_yz mean_w_yz+std_w_yz]);

w_xy = 0.1; % assume w_xy = 0.1

num = [w_xy*u_w_yz]; % numerator of G(z)

den = [1 -(u_w_y+u_w_z) u_w_y*u_w_z+u_w_yz*u_w_zy]; % denominator of G(z)

G = tf(num, den, 0.11); % discrete-time model with sampling period 0.11 hour

%Monte Carlo random sampling (100) of combinations of 4 uncertain parameters

%and the resulting pole-zero map

figure

pzmap(usample (G,100))

zgrid

%% Uncertain model Set 3

% y: Mdm2

% u: p53

clc

clear all;

close all;

mean_w_y = 0.8737;

std_w_y = 0.0830;

mean_w_z = 0.0998; % 0.3627 --> 0.0998

std_w_z = 0.0825;

mean_w_zy = 0.2662;

std_w_zy = 0.0343;

mean_w_yz = 0.6708; % 0.1656 --> 0.6708

std_w_yz = 0.1169;

u_w_y = ureal('u_w_y', mean_w_y, 'range', [mean_w_y-std_w_y mean_w_y+std_w_y]);

u_w_z = ureal('u_w_z', mean_w_y, 'range', [mean_w_z-std_w_z mean_w_z+std_w_z]);

u_w_zy = ureal('u_w_zy', mean_w_y, 'range', [mean_w_zy-std_w_zy mean_w_zy+std_w_zy]);

u_w_yz = ureal('u_w_yz', mean_w_y, 'range', [mean_w_yz-std_w_yz mean_w_yz+std_w_yz]);

w_xy = 0.1; % assume w_xy = 0.1

num = [w_xy*u_w_yz]; % numerator of G(z)

den = [1 -(u_w_y+u_w_z) u_w_y*u_w_z+u_w_yz*u_w_zy]; % denominator of G(z)

G = tf(num, den, 0.11); % discrete-time model with sampling period 0.11 hour

%Monte Carlo random sampling (100) of combinations of 4 uncertain parameters

%and the resulting pole-zero map

figure

pzmap(usample (G,100))

zgrid
